# S-Assimilation Influences in Carrageenan Biosynthesis Genes during Ethylene-Induced Carposporogenesis in Red Seaweed *Grateloupia imbricata*

**DOI:** 10.3390/md20070436

**Published:** 2022-06-29

**Authors:** Diana del Rosario-Santana, Rafael R. Robaina, Pilar Garcia-Jimenez

**Affiliations:** Departamento de Biologia, Facultad de Ciencias del Mar, Instituto de Estudios Ambientales y Recursos Naturales, Universidad de Las Palmas de Gran Canaria, E-35017 Las Palmas de Gran Canaria, Canary Islands, Spain; diana.delrosario@ulpgc.es (D.d.R.-S.); rafael.robaina@ulpgc.es (R.R.R.)

**Keywords:** carbohydrate sulfotransferase, carrageenan, galactose 1 phosphate uridyltransferase, galactose-6-sulfurylase, phosphoglucomutase, red algae

## Abstract

The synthesis of cell-wall sulfated galactans proceeds through UDP galactose, a major nucleotide sugar in red seaweed, whilst sulfate is transported through S-transporters into algae. Moreover, synthesis of ethylene, a volatile plant growth regulator that plays an important role in red seaweed reproduction, occurs through S-adenosyl methionine. This means that sulfur metabolism is involved in reproduction events as well as sulfated galactan synthesis of red seaweed. In this work we study the effects of methionine and MgSO_4_ on gene expression of polygalactan synthesis through phosphoglucomutase (PGM) and galactose 1 phosphate uridyltransferase (GALT) and of sulfate assimilation (S-transporter and sulfate adenylyltransferase, SAT) using treatment of ethylene for 15 min, which elicited cystocarp development in *Grateloupia imbricata*. Also, expressions of *carbohydrate sulfotransferase* and *galactose-6-sulfurylase* in charge of the addition and removal of sulfate groups to galactans backbone were examined. Outstanding results occurred in the presence of methionine, which provoked an increment in transcript number of genes encoding S-transporter and assimilation compared to controls regardless of the development stage of thalli. Otherwise, methionine diminished the transcript levels of *PGM* and *GALT* and expressions are associated with the fertilization stage of thalli of *G. imbricata*. As opposite, methionine and MgSO_4_ did not affect the transcript number of *carbohydrate sulfotransferase* and *galactose-6-sulfurylase*. Nonetheless, differential expression was obtained for sulfurylases according to the development stages of thalli of *G. imbricata*.

## 1. Introduction

**Figure 1:**
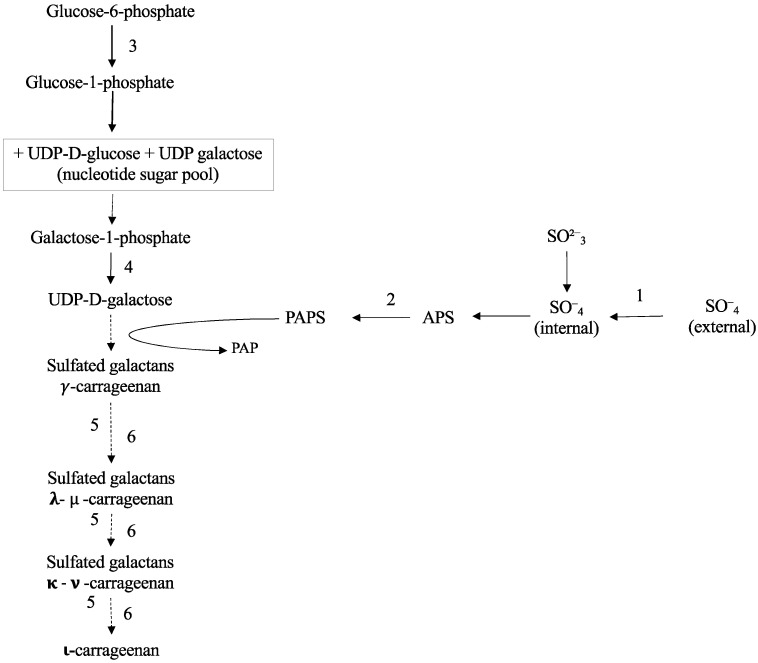
Schematic biosynthetic pathway for sulfate assimilation and synthesis of carrageenan with indication of enzymes studied in this work. PAPS, 3′phosphoadenosine-5′phosphosulfate; APS adenosine 5′-phosphosulfate; (1) S transporter; (2) sulfate adenylyltransferase; (3) phosphoglucomutase; (4) galactose 1 phosphate uridyltransferase; (5) carbohydrate sulfotransferase; (6), galactose-6-sulfurylase I, II.

Seaweeds are rich in sulfated galactans, accounting for over 60% of the carrageenan dry weight in red seaweed. Carrageenans are made of a backbone with linear chains of repeating D-galactose sugars and 3,6-anhydrogalactose units, with a different number and location of the sulfate groups attached. Sulfation and desulfation generate different carrageenan types such as κ-, ι-, and λ-carrageenan, which may even be found in different phases of the life cycle, albeit seaweeds can also contain hybrid carrageenans [1]. Sulfation takes place by the action of carbohydrate sulfotransferase and desulfation by galactose-6-sulfurylase (Figure 1), although its role is not fully clarified [2]. Galactose-6-sulfurylase catalyzes the conversion of μ-carrageenan into κ-carrageenan, but λ-carrageenan does not seem to be susceptible to its action [3,4]. Carrageenan type and the degree of sulfation render the thallus flexible, and differences in the strengths of carrageenans are related to algal development stage. Genes encoding carbohydrate sulfotransferase and galactose-6-sulfurylase have been associated at different development stages of red seaweed *Grateloupia imbricata* with the seasonal period [5].

Synthesis of cell-wall-sulfated galactans proceeds through UDP galactose, a major nucleotide sugar in red seaweed [6]. Sulfate is transported into algae through S-transporter and then activated by Sulfate adenylyltransferase (SAT). These reactions yield adenosine-5′-phosphosulfate (APS) that is phosphorylated to 3′phosphoadenosine-5′-phosphosulfate (PAPS); both are the source of sulfation of UDP galactose. Although little is known about what happens inside algae, UDP galactose can also be obtained reversibly from UDP-glucose and glucose 1-P by means of galactose 1 phosphate uridyltransferase (GALT). Phosphoglucomutase (PGM), on the other hand, operates in the conversion of glucose 6-P to glucose 1-P (Figure 1; [7]). Furthermore, biosynthesis of the hexose-phosphate pool addresses the polysaccharides synthesis as it occurs in the brown seaweed *Saccharina japonica* [8] and in the unicellular red alga *Galdieria sulphuraria* [7].

Moreover, a central and important role in sulfur metabolism in algae and plants is played by S-adenosylmethionine (SAM; [9]), which has as its precursor as the sulfur-containing amino acid methionine. SAM is, in turn, the precursor of ethylene, a plant growth regulator with conspicuous functions in red seaweed reproduction. In particular, a treatment of ethylene for 15 min elicited cystocarps in an early stage of development in the carragenophytic red seaweed *Grateloupia imbricata* (cystocarp disclosure; [10,11,12,13]).

**Figure 2:**
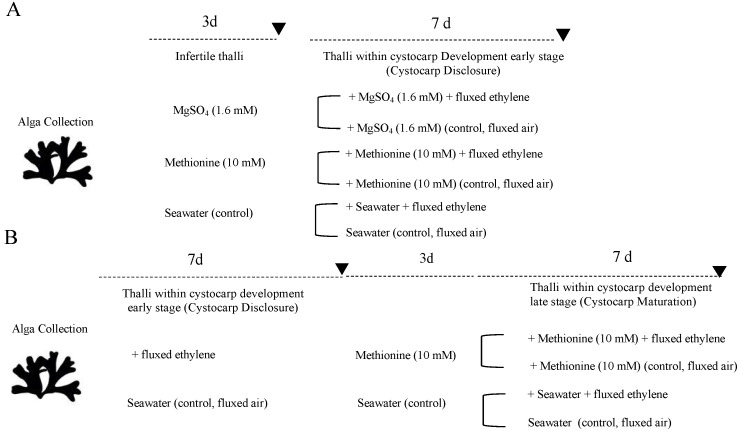
Scheme showing timeline for determination of gene expression (bold arrowhead) in *Grateloupia imbricata*: (**A**) in infertile thalli treated with methionine and MgSO_4_ for 3 days, and when early stages of cystocarp development were elicited after a 15 min ethylene treatment (end time: 10 days); (**B**) in thalli within early stages of cystocarp development after 15 min ethylene treatment at 7 days, and when thalli reached late stages of cystocarp development after addition of methionine and fluxed ethylene (endtime 17 days). Note that different controls are indicated.

It was hypothesized that carrageenan synthesis could be affected by S-compounds, namely, sulfate and methionine, in ethylene-induced stages of development. Thus, under a working model with the carragenophytic *G. imbricata* and a set time period to elicit cystocarp disclosure by supplying exogenous ethylene (Figure 2), our aim was to analyze the expressions of genes involved in processes of S-transport and assimilation, and in synthesis of UDP-galactose as a precursor of carrageenan synthesis through *PGM* and *GALT*. Furthermore, genes responsible for sulfation (*carbohydrate sulfotransferase*) and desulfation (*galactose-6-sulfurylase I, II*) of galactan backbone of cell-wall polysaccharides were analyzed. Monitoring of cystocarp disclosure is carried out with the marker gene of reproduction of red seaweed, *ornithine decarboxylase* (*ODC*; [14])

## 2. Results

A candidate gene of reproduction in red seaweed, *ornithine decarboxylase* (*ODC*), showed differential expression for infertile thalli (2.75 ± 2.1 × 10^−2^ copies μL^−1^) compared to that for thalli within early-stage cystocarp development (1.3 ± 1.8 × 10^−2^ copies μL^−1^), as expected [10].

### 2.1. Assimilation of S-Source

**Figure 3:**
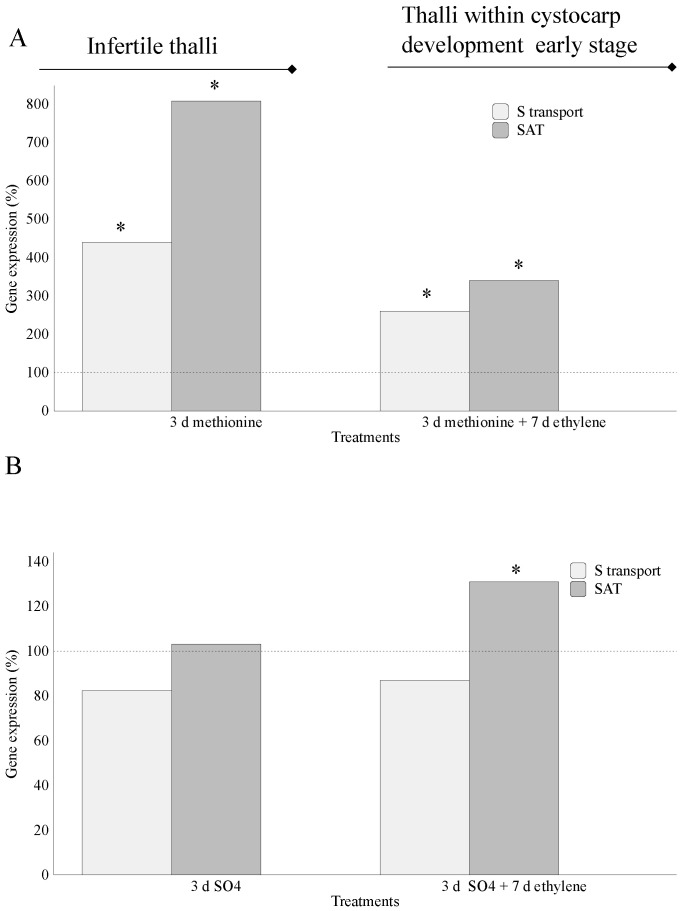
Expression of genes that encode sulfate transporter (S-transporter) and sulfate adenylyltransferase (SAT) at day 3 (addition of S-source) and day 10 (S-source plus fluxed ethylene) in thalli of *Grateloupia imbricata* (as in experiment A in Figure 2). Expression was analyzed in (**A**) infertile thalli after addition of exogenous methionine and in thalli that reached early-stage cystocarp development after methionine plus ethylene; and (**B**) infertile thalli after addition of exogenous MgSO_4_ and in thalli that reached early-stage cystocarp development after MgSO_4_ plus ethylene. Expression (copies μL^−1^) is shown as a percentage relative to expression in untreated thalli at day 3 and day 10 for methionine and MgSO_4,_ respectively (100%, dashed horizontal line). For methionine, infertile thalli gene expression (i.e., 100%), *S-transporter* = 1.55 ± 3.35 × 10^−3^ and *SAT* = 1.3 ± 2.15 × 10^−3^. In thalli within early-stage cystocarp development (10 days) gene expression (i.e., 100%), *S-transporter* = 4.0 ± 6 × 10^−4^ and *SAT* = 2.1 ± 7 × 10^−4^. For MgSO_4_, *S-transporter* = 1.55 ± 5 × 10^−5^ and *SAT* = 1.3 ± 5.5 × 10^−5^ in infertile thalli. In thalli within early-stage cystocarp development, *S-transporter* = 1.7 ± 5 × 10^−5^ and SAT = 1.9 ± 1.05 × 10^−4^. * means significant difference (*p* < 0.01) between infertile thalli and its respective control at day 3 and between thalli within early-stage cystocarp development and its control at day 10.

Thalli previously treated with methionine, as an external S-source, showed significant differences for gene-encoding S-transporter and SAT compared to their controls. Furthermore, *S-transporter* (440%) and *SAT* (807.7%) gene expressions were higher in infertile thalli compared to those in thalli within early-stage cystocarp-development (260% for *S-transporter* and 340% for *SAT*; Figure 3A). No significant differences were observed in thalli treated with MgSO_4_, with the exception of *SAT* expression in thalli within early-stage cystocarp development (131%; Figure 3B).

### 2.2. Carrageenan Synthesis

**Figure 4:**
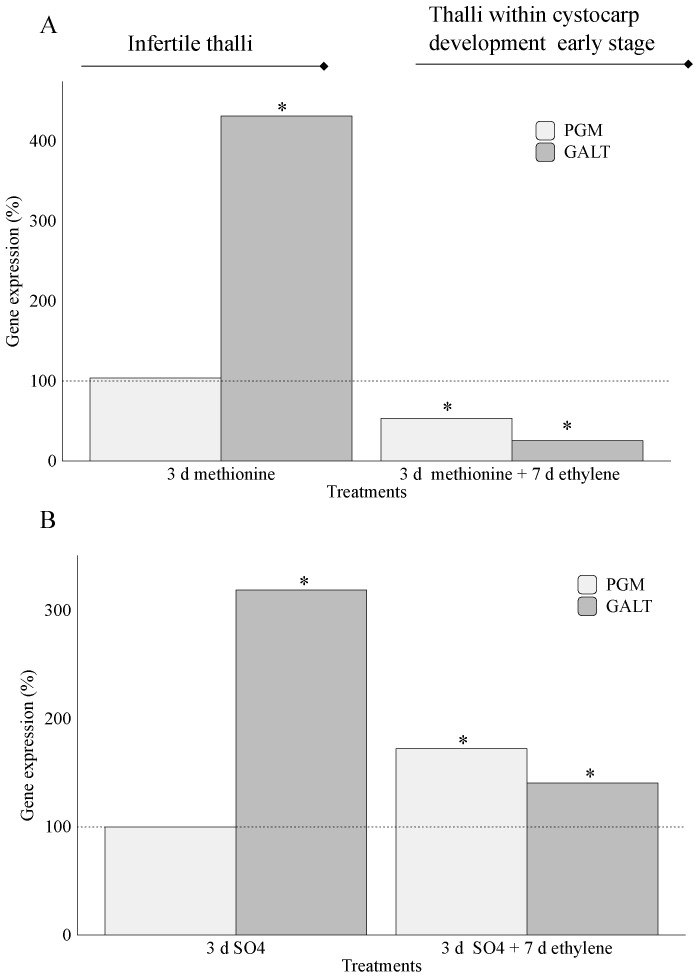
Expression of genes that encode phosphoglucomutase (PGM) and galactose 1 phosphate uridyltransferase (GALT) at day 3 (addition of S-source) and day 10 (S-source plus fluxed ethylene) in thalli of *Grateloupia imbricata* (as in experiment A in Figure 2). Expression was analyzed in: (**A**) infertile thalli after addition of exogenous methionine and in thalli that reached early-stage cystocarp development after methionine plus ethylene, and (**B**) infertile thalli after addition of exogenous MgSO_4_ and in thalli that reached early-stage cystocarp development after MgSO_4_ plus ethylene. Expression (copies μL^−1^) is shown as a percentage relative to expression in untreated thalli at day 3 and day 10 for methionine and MgSO_4,_ respectively (100%, dashed horizontal line). For methionine, infertile thalli gene expression (i.e., 100%) is *PGM* = 1.25 ± 1.65 × 10^−3^ and *GALT* = 1.6 ± 5 × 10^−5^. In thalli within early-stage cystocarp development, gene expression (i.e., 100%) is *PGM* = 2.25 ± 1.7 × 10^−3^ and *GALT* = 25 ± 6.35 × 10^−3^. For MgSO_4_, *PGM* = 1.25 ± 1.7 × 10^−3^, and *GALT* = 1.6 ± 1.45 × 10^−3^. In thalli within early-stage cystocarp development, *PGM* = 0.725 ± 2.05 × 10^−3^ and *GALT* = 1.6 ± 1.45 × 10^−3^. * means significant difference (*p* < 0.01) between infertile thalli and its respective control at day 3 and between thalli within cystocarp development early stage and its control at day 10.

An evaluation of the expression levels of precursors of carrageenan synthesis showed that *galactose 1 phosphate uridyltransferase* (*GALT*) was significantly overexpressed in infertile thalli cultivated in the presence of methionine (431.3%) and SO_4_^2−^ (319%; Figure 4A,B). In thalli that reached early-stage cystocarp development, overexpression of *GALT* was only reported in the SO_4_^2−^ treatment (140.6%; Figure 4B). By contrast, non-significant expression for *phosphoglucomutase* (*PGM*) transcripts occurred in infertile thalli cultivated in the presence of methionine (Figure 4A) and SO_4_^2−^ (Figure 4B) and in thalli cultivated with methionine and fluxed with ethylene (Figure 4A).

**Figure 5:**
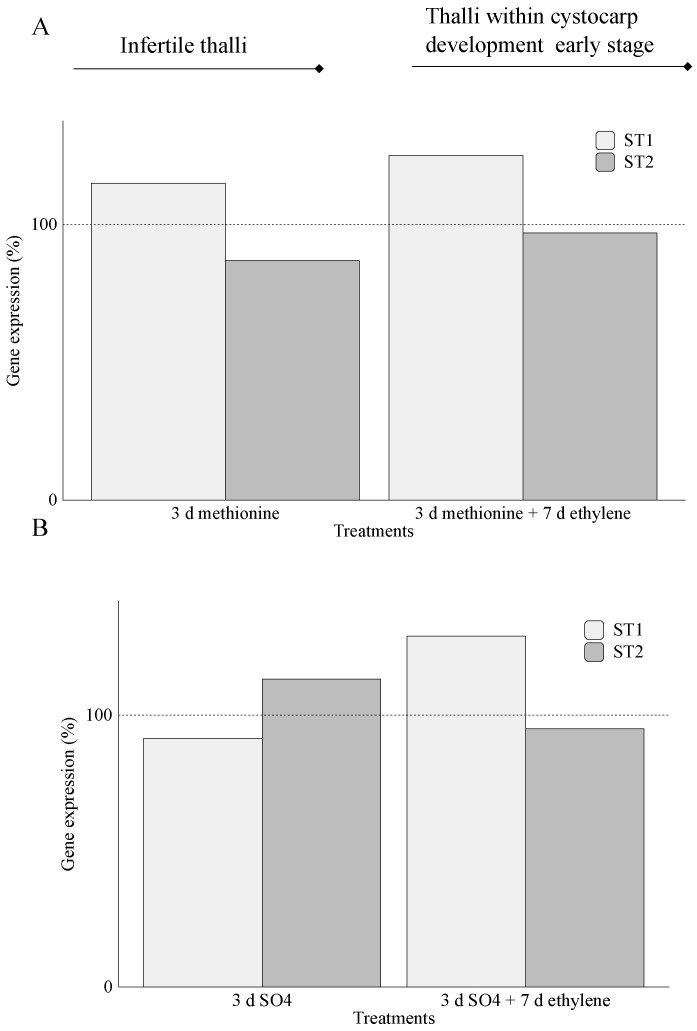
Expression of genes that encode carbohydrate sulfotransferase (ST1 and ST2) at day 3 (addition of S-source) and day 10 (S-source plus fluxed ethylene) in thalli of *Grateloupia imbricata* (as in experiment A in Figure 2). Expression was analyzed in (**A**) infertile thalli after addition of exogenous methionine and in thalli that reached early-stage cystocarp development after methionine plus ethylene, and (**B**) infertile thalli after addition of exogenous MgSO_4_ and in thalli that reached early-stage cystocarp development after MgSO_4_ plus ethylene. Expression (copies μL^−1^) is shown as a percentage relative to expression in untreated thalli at day 3 and day 10 for methionine and MgSO_4,_ respectively (100%, dashed horizontal line). For methionine, infertile thalli gene expression (i.e., 100%) is *ST1* = 1.75 ± 1.45 × 10^−3^ and *ST2* = 1.5 ± 5 × 10^−4^. In thalli within early-stage cystocarp development, gene expression (i.e., 100%) is *ST1* = 6.4 ± 1.1 × 10^−4^ and *ST2* = 4.9 ± 4.5 × 10^−4^. For MgSO_4_, *ST1* = 1.75 ± 1.0 × 10^−2^ and *ST2* = 1.5 ± 5 × 10^−5^. In thalli within early-stage cystocarp development, *ST1* = 0.8 ± 5 × 10^−5^ and *ST2* = 1.7 ± 5 × 10^−5^.

The transcript expression levels of each of two gene sequences encoding carbohydrate sulfotransferase (ST1 and ST2) exhibited similar behavior for thalli treated with methionine (115% and 80% for *ST1* and *ST2*, respectively) and those in methionine plus ethylene (125% for *ST1* and 97% for *ST2*; Figure 5A). Furthermore, non-significant differences were observed in thalli treated with SO_4_^2−^ regardless of stage of development, i.e., 91.4% and 129% for *ST1* in infertile thalli and thalli at early-stage cystocarp development, respectively, and 113.3% and 95% for *ST2* for the same stages (Figure 5B).

**Figure 6:**
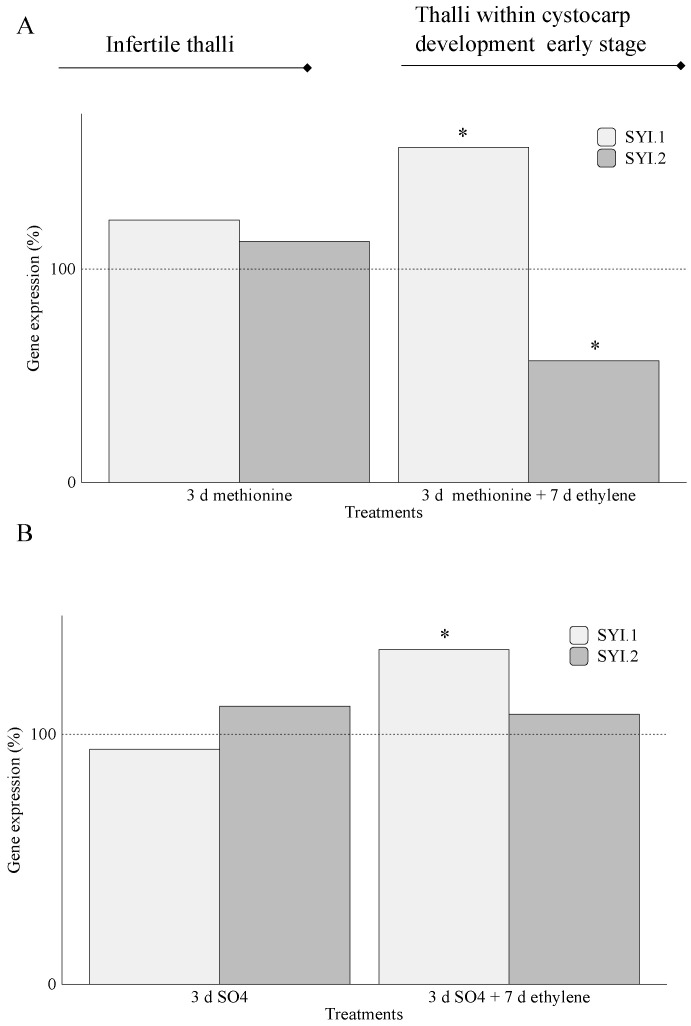
Expression of genes that encode galactose-6-sulfurylase type I (SYI.1 and SYI.2) at day 3 (addition of S-source) and day 10 (S-source plus fluxed ethylene) in thalli of *Grateloupia imbricata* (as in experiment A in Figure 2). Expression was analyzed in (**A**) infertile thalli after addition of exogenous methionine and in thalli that reached early-stage cystocarp development after methionine plus ethylene, and in (**B**) infertile thalli after addition of exogenous MgSO_4_ and in thalli that reached early-stage cystocarp development after MgSO_4_ plus ethylene. Expression (copies μL^−1^) is shown as a percentage relative to expression in untreated thalli at day 3 and day 10 for methionine and MgSO_4,_ respectively (100%, dashed horizontal line). For methionine, infertile thalli gene expression (i.e., 100%) is *SYI.1* = 1.0 ± 1.1 × 10^−4^ and *SYI.2* = 2 ± 1.05 × 10^−3^. In thalli within early-stage cystocarp development, gene expression (i.e., 100%) is *SYI.1* = 2 ± 6.0 × 10^−4^ and *SYI.2* = 1.9 ± 1.15 × 10^−4^. For MgSO_4_, *SYI.1* = 1.0 ± 5.5 × 10^−4^ and *SYI.2* = 20 ± 1.85 × 10^−3^. In thalli within early-stage cystocarp development, *SYI.1* = 2.0 ± 1.55 × 10^−3^ and *SYI.2* = 1.9 ± 5 × 10^−5^. * means significant difference (*p* < 0.01) between infertile thalli and its respective control at day 3 and between thalli within cystocarp development early stage and its control at day 10.

In infertile thalli, two gene sequences encoding galactose-6-sulfurylase type I (SYI.1 and SYI.2) showed non-significant expression differences when transcript levels of *SYI.1* and *SYI.2* were compared to their controls (Figure 6A,B). In thalli within early-stage cystocarp development, expression levels of *SYI.1* showed significant differences regardless of whether thalli were treated with methionine (Figure 6A) or with MgSO_4_ (Figure 6B). In particular, *SYI.1* expression was higher than *SYI.2* in thalli treated with methionine plus ethylene (Figure 6A) and in thalli treated with MgSO_4_ plus ethylene (Figure 6B).

**Figure 7:**
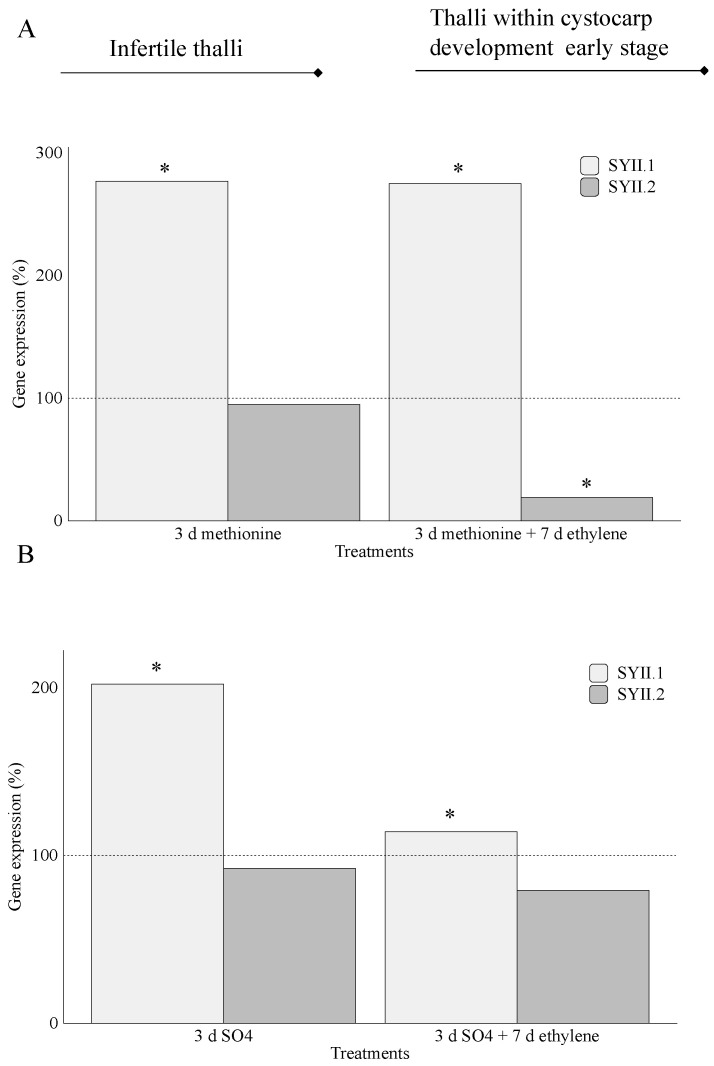
Expression of genes that encode galactose-6-sulfurylase type II (SYII.1 and SYII.2) at day 3(addition of S-source) and day 10 (S-source plus fluxed ethylene) in thalli of *Grateloupia imbricata* (as in experiment A in Figure 2). Expression was analyzed in (**A**) infertile thalli after addition of exogenous methionine and in thalli that reached early-stage cystocarp development after methionine plus ethylene, and (**B**) infertile thalli after addition of exogenous MgSO_4_ and in thalli that reached early-stage cystocarp development after MgSO_4_ plus ethylene. Expression (copies μL^−1^) is shown as a percentage relative to expression in untreated thalli at days 3 and 10 for methionine and MgSO_4,_ respectively (100%, dashed horizontal line). For methionine, infertile thalli gene expression (i.e., 100%) is *SYII.1* = 0.9 ± 5.0 × 10^−5^ and *SYII.2* = 1.95 ± 9.0 × 10^−3^. In thalli within early-stage cystocarp development, gene expression (i.e., 100%) is *SYII.1* = 2.5 ± 1.05 × 10^−3^ and *SYII.2* = 1.85 ± 9.5 × 10^−6^. For MgSO_4_, *SYII.1* = 0.9 ± 5.0 × 10^−5^ and *SYII.2* = 1.95 ± 4.5 × 10^−5^. In thalli within early-stage cystocarp development, *SYII.1* = 2.5 ± 4.35 × 10^−6^ and *SYII.2* = 1.85 ± 5.5 × 10^−4^. * means significant difference (*p* < 0.01) between infertile thalli and its respective control at day 3 and between thalli within early-stage cystocarp development and its control at day 10.

Furthermore, *SYII.1* was overexpressed compared to *SYII.2*, both in thalli treated with methionine (Figure 7A) and in those treated with MgSO_4_ (Figure 7B), regardless of development stage (Figure 7A,B). Remarkably, drastic down expression of *SYII.2* (19%) occurred in thalli within early-stage cystocarp development in the presence of methionine (Figure 7A). Moreover, in thalli treated with MgSO_4,_ high levels of *SYII.1* appeared in infertile thalli (202%) when compared to thalli in early-stage cystocarp development (114%; Figure 7B).

## 3. Discussion

The exploitation of raw material from seaweed, such as carrageenan, greatly depends on the quality of sulfated galactans (SG). According to sulfation degrees, different types of carrageenans can be synthetized, which then shape gel networks. Moreover, the sulfation and desulfation degree of galactan backbone of SG is associated with alterations in thalli development and cystocarp maturation in red seaweeds [5]. Given that carposporogenesis in *G. imbricata* is an asynchronous process, in which the developmental stage of cystocarps is difficult to determine accurately, the pursuit of the *ODC* gene confirmed a down expression as development and maturation of reproductive structures (cystocarps) occurred [12,13,15]. In addition, the expression for each of the genes in thalli treated with an S-source and those also fluxed with ethylene was compared to corresponding untreated thalli at each time point (i.e., 3 and 10 days) to avoid bias (Figure 2A).

The transport, activation, and assimilation of sulfate require fine control through different genes that are regulated according to external signals; sulfur availability; and balanced interactions between the N, C, and S pathways in higher plants [16]. Sulfate uptake is controlled through demand-driven regulation such as sulfate transporter, which can be repressed when an amount of reduced sulfur is available for plants [17], which seems not to be the case in *G. imbricata*. In this study, exogenous addition of methionine, a reduced source of S, provoked an increment in the transcript number (in copies μL^−1^) of genes encoding S-transporter and assimilation (*S-transporter* and *SAT*) (Figure 3A). Furthermore, although expressions were lower in ethylene-induced fertile thalli than in those from infertile thalli, *S-transporter* and *SAT* were always overexpressed compared to controls (Figure 3A). Certainly, increments in basal levels of transcripts allow us to infer that methionine may be stored and further favor overexpression of transcripts for *S-transporter* and assimilation into S-compounds differentially from gene expressions led by MgSO_4_. Otherwise, MgSO_4_ might make transport and assimilation systems insensitive as there are non-limiting levels of sulfate in the seawater (Figure 3B; [18]).

Experimentation with two S sources, methionine and MgSO_4_, in the study model system of *G. imbricata* allows greater insight into the involvement of S in algal metabolism. Indeed, metabolism is shaped by genes, but metabolic machinery is affected by changes in the concentration of metabolites, which, in turn, can act as substrates and cofactors for post-translational modifications. Although recognized, this fact could be exemplified with the pool of methyl donor S-methyl methionine (SMM) and S-adenosyl methionine (SAM), synthetized from methionine, as SAM/SMM can fluctuate in concentration, limit activity of methyltransferases, and influence regulation of gene expression in several organisms [19,20]. Our results open a door to study whether genes in charge of transport and assimilation can be regulated by a pool of sulfur organic compounds in algae.

Outstandingly, the expression of genes encoding proteins in charge of S-transport was uncorrelated with that for S-assimilation. Little is known about the type of S-transporters, their cell location, and enzyme isomorphs in algae [21]. Sulfate transporters can be expressed in different parts of a plant [22] and could potentially work in the transport of different reduced forms of sulfur [23]. In addition, many other transporters and forms of organic sulfur can be used by organisms, as these S-forms are also products of the assimilation pathway. In thalli of *G. imbricata*, transcript levels of the gene-encoding S-transporter showed lower expressions than those of the *SAT* gene. Remarkably, these expressions displayed maximum differences in infertile thalli cultured in methionine for 3 days (440% for *S-transporter* and 808% for *SAT* above their respective controls; Figure 3A). Likewise, thalli within early-stage cystocarp development induced by ethylene showed transcript expression for *S-transporter* of 260% and 340% for *SAT* (Figure 3A). Unlike genes that have the same trend in methionine and MgSO_4,_ these results seem to reaffirm a conspicuous regulation of these genes with respect to methionine (Figure 3A) compared to that in the presence of MgSO_4_ (Figure 3B).

**Figure 8:**
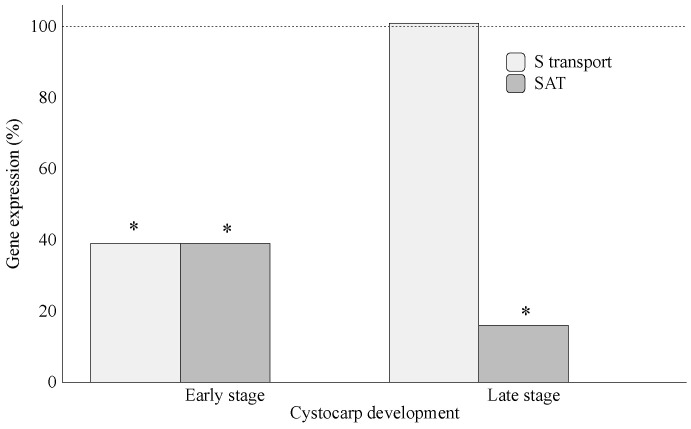
Expression of genes that encode sulfate transporter (*S-transporter*) and sulfate adenylyltransferase (*SAT*) after an ethylene flux in thalli of *Grateloupia imbricata* (Experiment B in Figure 2). Expression was analyzed in thalli within early-stage cystocarp development at day 7 (in the left), and in thalli within cystocarp development late stage at day 17 (in the right). Expression (copies μL^−1^) is shown as a percentage relative to expression in untreated thalli at day 7 and 17 (100%, dashed horizontal line). In thalli within early-stage cystocarp development (day 7), gene expression (i.e., 100%) is *S-transporter* = 3.05 ± 6 × 10^−4^ and *SAT =* 1.8 ± 7 × 10^−4^. In thalli within late-stage cystocarp-development (17 day), *S-transporter* = 2.02 ± 6 × 10^−4^ and *SAT =* 0.98 ± 1.1 × 10^−5^. * means significant difference (*p* < 0.01) between thalli within early-stage cystocarp development and its respective control at day 7 and between thalli within late-stage cystocarp development and its control at day 17.

It is known that the synthesis of S-containing amino acids and intermediates requires sulfide, which favors cysteine and methionine synthesis [24,25]. In particular, methionine is a precursor of ethylene synthesis [26], and the latter has been described as improving S acquisition [27]. Furthermore, methionine is used to synthetize S-methyl methionine (SMM) and S-adenosyl methionine (SAM; [28]). Otherwise, the SAM level is controlled by SMM, whereas SAM is the precursor for the biosynthesis pathway of ethylene, which has a main role in red seaweed reproduction [13]. Therefore, it would be reasonable to infer that SAM and SMM availability might increase the demand for supplying reduced S, thus favoring over-expression of gene-encoding S-assimilation in infertile thalli (808% *SAT*) and in thalli within early cystocarp development stages (340% *SAT*) (Figure 3A). It would remain to be solved whether diminished gene expressions (in percentage) of *S-transporter* and assimilation in thalli within early-stage cystocarp development (i.e., thalli cultured in methionine plus ethylene) compared to infertile thalli (cultured only with methionine) are due to S availability, as methionine is being supplied exogenously, or due to changes in reproductive stages elicited by ethylene. Thus, trying to solve this issue, a new assay (Figure 2B) revealed that gene expression of *SAT* decreased drastically (16% *SAT* expression) in *G. imbricata* thalli within cystocarp development late stages (ethylene-induced cystocarp maturation), while *S-transporter* maintained basal level (approx. 100% for *S-transporter*; Figure 8). Broadly, these results show that (i) *S-transporter* expression tends to sustain alongside transition from infertile to fertilization, and different stages occur as methionine may be stored in the S-organic pool; and (ii) S-assimilation, to convert methionine to SAM-activated form, does not seem to be demanded in late stages of ethylene-induced cystocarp development (Figure 8). Moreover, up-expression of *SAT* in infertile and early-stage cystocarps indicate that S-source could be stored for further assimilation in a specific zone of thalli where S-source is needed, and assimilation will take place specifically. Thus, in thalli within early-stage cystocarp development, i.e., in the presence of ethylene plus methionine (Figure 3A), the overexpression of *SAT* could be required to sufficiently activate sulfate for generating reduced forms of S and further to induce cystocarp development (Figure 3A,B). Once induced, *SAT* diminishes as it occurs in late early stages (Figure 8). Evidence of high transcript levels of *SAT* have been reported in higher plants according to different tissues such as growing leaves and root tips of *Arabidopsis* [29,30]. Furthermore, despite thalli simplicity in red seaweeds, differential gene expressions could be conceived as they have been previously reported according to both the reproductive stage and the apical and basal zone of thalli in *G. imbricata* (e.g., *ODC* reproduction marker gene [31,32]). With our framework of well-defined cystocarp development stages, this study opens a network to gain clearer and more accurate insight into the metabolism of S in seaweed reproduction, the role of ethylene as a trigger of algal reproduction and its involvement in SAM regulation as an activated form of methionine, and into the biosynthesis of cell-wall sulfate polysaccharides in seaweeds. Modifications of development stages of thalli were always verified through changes in transcript levels of the reproduction marker gene in red seaweeds, *ODC*, which decreased alongside cystocarp development (copies μL^−1^ for *ODC* in infertile thalli, 2.75 ± 2.1 × 10^−2^; within early-stage cystocarp development, 1.3 ± 1.8 × 10^−2^; and within cystocarp development late stage, 0.99 ± 1.0 × 10^−3^).

**Figure 9:**
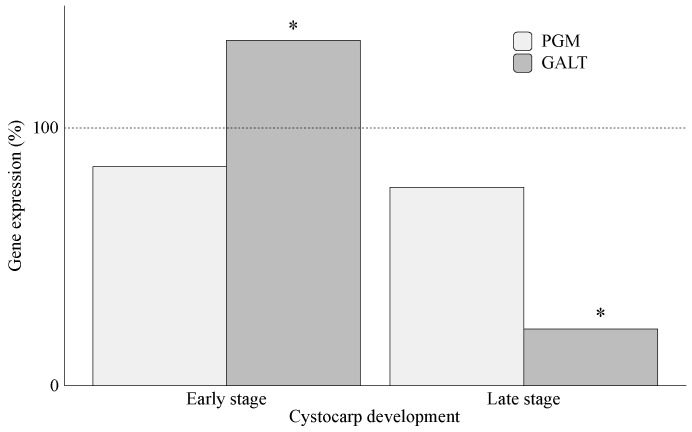
Expression of genes that encode proteins phosphoglucomutase (PGM) and galactose-1-phosphate uridyltransferase (GALT) after ethylene flux in thalli of *Grateloupia imbricata* (Experiment B in Figure 2). Expression was analyzed in thalli within early-stage cystocarp development at day 7 (in the left), and in thalli within cystocarp development late stage at day 17 (in the right). Expression (copies μL^−1^) is shown as a percentage relative to expression in untreated thalli at day 7 and day 17 (100%, dashed horizontal line). In thalli within early-stage cystocarp development (7 d), gene expression (i.e., 100%) is *PGM* = 1.25 ± 1.1 × 10^−3^ and *GALT* = 8.9 ± 2.13 × 10^−4^. In thalli within late-stage cystocarp development (14 d), *PGM* = 1.19 ± 0.89 × 10^−4^ and *GALT* =1.85 ± 1.1 × 10^−4^. * means significant difference (*p* < 0.01) between thalli within early-stage cystocarp development and its respective control at day 7 and between thalli within cystocarp development late stage and its control at day 17.

Once the sulfur is assimilated, the resulting product PAPS is used for the sulfation of UDP galactose. Additionally, UDP galactose can be also obtained through the conversion of galactose by means of galactose 1 phosphate uridyltransferase (GALT), while phosphoglucomutase (PGM) is in charge of conversion of glucose 6-P to 1-P (Figure 1). Although PGM’s role in algae has been neglected, it has been reported that this enzyme can be activated by bivalent cations such as Mg^2+^ in the brown seaweed *Saccharina japonica* [8]. In this way, high levels of transcripts of *PGM* could be expected in thalli of *G. imbricata* cultivated in the presence of MgSO_4_. Nonetheless, *PGM* overexpression only occurs in thalli within early-stage cystocarp development (172%; Figure 4B). This could be explained as hexose pool would increase because they are used as glucose 1-P is a precursor for polysaccharide synthesis, i.e., mucilage synthesis for spores protecting. Although no evidence has been reported in algae, conversion of glucose from 6-P to 1-P by PGM has been described as crucial for sporophyte and gametophyte development of Arabidopsis [33]. It is striking what occurs in thalli cultured in the presence of methionine (Figure 4A). A down expression of *PGM* (nearly 53% *PGM* expression compared to its control; Figure 4A) might indicate a reduction to mobilize hexoses, avoiding polysaccharide synthesis in the early stage of cystocarp development. This result would be in accordance with *PGM* expression that compares early (elicitation) and late (maturation) stages of cystocarp development (Figure 2B and Figure 9). In this case, *PGM* expression was unaltered in *G. imbricata* thalli within the early (percentage of gene expression, 85%) and late (percentage of gene expression, 77%) stages of cystocarp development as shown in Figure 9.

Furthermore, glucose 1-P is used to render nucleotide sugars, such as UDP glucose. These nucleotide sugars are substrates of cell-wall synthesis and depend on the growth stage of the tissue [34]. The *GALT* gene-encoding protein responsible for one of the biochemical steps that produces these precursors has been described in the red seaweed *Gracilaria changii*, where an abundance of transcripts of *GALT* has been correlated to synthesis of sulfated polysaccharides [35]. Therefore, *GALT* expression could be associated with the fertilization stage of thalli of *G. imbricata*. Thus, in *G. imbricata*, reduced expression of *GALT*, reported in the early stage of development compared to infertile thalli, seems to show fluctuations in the composition of wall-galactans to locate reproductive structures in thalli within early stage cystocarp development (Figure 4A,B). Indeed, *GALT* was significantly reduced in the late-development stages (mature cystocarps; Figure 9), which confirms alterations in biosynthesis of different wall-galactans.

Although biosynthetic pathways for carrageenan have not been completely elucidated in red seaweeds, UDP-galactose is deemed a precursor for cell-wall sulfated galactans biosynthesis through the addition and removal of sulfate groups from C-backbone [36,37]. The *G. imbricata* expression of two annotated *carbohydrate sulfotransferases* (*ST1* and *ST2*) seems to be constitutive for *G. imbricata*, as non-significant differences between transcript levels of *ST1* and *ST2* were encountered compared to those from the control and considering both S source (methionine vs. MgSO_4_) and cystocarp development stage of thalli (infertile thalli without cystocarp vs. thalli within early-stage cystocarp development; Figure 5A,B). Moreover, it can be assumed that *ST* gene expressions, which encode proteins in charge of adding sulfate groups to cell-wall galactan, might be a consequence of carrageenan synthesis and also neutral polysaccharides, which are a constituent of mucilage in red algae [38]. No changes in *ST* expression were observed in *G. imbricata* when through field sampling; *ST1* and *ST2* were also unaltered in infertile, fertilized (well-developed cystocarps), and fertile (fully developed cystocarps) thalli [5].

The transcript levels of two annotated *galactose-6-sulfurylase* type I (*SYI.1* and *SYI.2*) showed a time-regulated behavior as *SYI.1* expression was higher in early-stage cystocarp development than those in infertile thalli in the presence of both exogenous methionine and MgSO_4_ (Figure 6A,B). Likewise, our data show that *SYI.1* also displays a fertilization-specific expression, as differences were encountered for *SYI.1* compared to *SYI.2* for these early stages (Figure 6A,B). This differential behavior of *SYI.1* and *SYI.2* was also reported when *SYI* transcript levels were analyzed in fertilized thalli of *G. imbricata* from field sampling [5]. Hence, it is worthwhile to suggest that, firstly, a time-gene regulation of two *galactose-6-sulfurylase* type I could be possible through the synthesis of specific transcription factors of *SYI.1* and *SYI.2*. Secondly, these genes, encoding proteins in charge of removing sulfate groups from sulfated galactans, would be working to soften and further support reproductive structures (cystocarps) in thalli, as occurred in those fertilized and fertile thalli of *G. imbricata* [5].

Considering *galactose-6-sulfurylase* type II, the *SYII.1* expression does not seem to be related to the development stage, as *SYII.1* was overexpressed in both infertile thalli and thalli within early-stage cystocarp development and regardless of S-source (Figure 7A,B). *SYII.1* gene expression was higher than that for *SYII.2* (Figure 7A,B). Overall, the data suggest a differential role for two sulfurylases type II. Thus, it is tempting to guess that if *SYII.1* is dependent on the reproductive stage, the alteration of expressions of *SYII.1*, and presumably of *SYII.2*, could be because genes are encoding different proteins that remove sulfate groups on multiple intermediates of sulfated-polysaccharide biosynthesis. Interestingly, *Grateloupia* sp. have been reported as mainly containing hybrid carrageenan at different rates, where κ- is the more prominent and ι- would be present to a lesser extent [39]. Thus, different expressions of *SYII* (1 and 2) could be associated with conversion from μ- to κ-carrageenans and to that from κ- to ι-carrageenans. Taking into account that maximum expression corresponds to *SYII.1* and the prevailing fraction is made of κ-carrageenans in *Grateloupia*, *SYII.1* may act in the conversion from μ- to κ-carrageenans and *SYII.2* from κ- to ι-carrageenans. The occurrence of carrageenan types and differential gene expression for *SYII* suggests the hypothesis that there could also be development stage-specific roles for these sulfurylases. This opens a path to study whether *galactose-6-sulfurylase* annotated in *G. imbricata* transcriptome works on multiple intermediates of sulfated-polysaccharide biosynthesis as proposed.

In summary, the results showed an increment in transcript number of genes encoding S-transporter and assimilation compared to controls regardless of the development stage of thalli in the presence of methionine. Otherwise, methionine diminished transcript levels of *PGM* and *GALT* but gene expressions are associated with the fertilization stage of thalli of *G. imbricata*. As opposite, methionine and MgSO_4_ did not affect the transcript number of *carbohydrate sulfotransferase* and *galactose-6-sulfurylase* (i.e., when UDP galactose is rendered, Figure 1). Nonetheless, differential expression was obtained for sulfurylases according to the development stages of thalli of *G. imbricata*. 

## 4. Materials and Methods

### 4.1. Plant Material and Culture Conditions

Infertile thalli from the carragenophytic *G. imbricata* were collected along the northeast coast of Gran Canaria in the Canary Islands. Thalli were placed in 500 mL vessels (3 g per vessel) and cultivated separately with two S-sources, methionine (10 mM) and magnesium sulfate (1.6 mM; MgSO_4_), for 3 days (Figure 2A). When proceeding, cystocarp development was elicited with ethylene ([10]; 99.9% purity, Carburos Metálicos SA, Barcelona, Spain), which was applied to the 500 mL sealed vessels for 15 min at a flow rate of 0.5 l min^−1^. Thalli continued to be cultivated for 7 days. Infertile thalli fluxed with ethylene reached early-stage cystocarp development as expected (day 10; [10]; Figure 2A). Thalli were maintained at 20 ± 2 °C under an 18 h light (30 µmol photons m^−2^·s^−1^): 6 h dark photoperiod in a growth chamber.

### 4.2. Changes in Gene Expression According to S-Source and to Elicitation of Cystocarps by Ethylene

The effect of type of sulfur source on assimilation of S-source and carrageenan synthesis was evaluated in *G. imbricata* thalli on the 3rd and 10th day as detailed above. Gene expression of the S-assimilation pathway i.e., *sulfate transporter (S-transporter)* and *Sulfate adenylyltransferase (SAT)* was determined (Figure 1). For carrageenan synthesis, genes such as *phosphoglucomutase* (*PGM*) and *galactose 1 phosphate uridyltransferase* (*GALT*)*,* two contigs annotated as *galactose-6-sulfurylase* type I (*SYI.1* and *SYI.2*), two contigs of *galactose-6-sulfurylase* type II (*SYII.1* and *SYII. 2*), and two contigs of *carbohydrate sulfotransferase* (*ST1* and *ST2*) were examined (Figure 1). Ornithine decarboxylase expression (*ODC*; [14]) as a marker gene of reproduction of red seaweed was also analyzed.

Thalli exposed to air flux instead of ethylene under the same experimental conditions were used as controls. Control (untreated) samples were cultured and processed in parallel. All samples were assayed in triplicate with two independent replicates for each experiment. At the end of the periods, samples of the thalli were frozen at −80 °C until the isolation of RNA.

### 4.3. RNA Extraction

The total RNA was separately extracted from the upper half regions (100 mg) of thalli using 1 mL Tri-Reagent (Sigma, St. Louis, MO, USA) according to the manufacturer’s instructions. The isolated RNA samples were individually suspended in 20 μL of 1 M Tris-HCl (pH 8), 0.5 M EDTA, and treated with DNase (1 U. mg^−1^, Promega, Madison, WI, USA) to destroy contaminating DNA. Total RNA was quantified using a TrayCell cuvette and Beckman Coulter DU 530 spectrophotometer. Next, extracted RNA from each sample (~1 μg) was reverse transcribed in the presence of oligo (dT) and primers with randomLy generated sequences from an iScript cDNA synthesis kit (Bio-Rad, Hercules, CA, USA). The reverse transcription procedure was carried out at 25 °C for 5 min, 42 °C for 30 min, and 85 °C for 5 min. The integrity of the cDNA was validated using a NanoDrop spectrophotometer (Thermo Fisher Scientific, Waltham, MA, USA). The products were kept at 4 °C until used.

### 4.4. Droplet Digital PCR (ddPCR) Primers and Protocol Implementation

For quantification of each target transcript by ddPCR, QX200 ddPCR EvaGreen Supermix (Bio-Rad) was used according to the manufacturer’s instructions. Briefly, for each sample, a PCR reaction mix (final volume, 20 μL) was prepared containing 1.5 μL of cDNA, 10 μL of QX200 ddPCR EvaGreen Supermix, and 0.22 μL of each primer (10 μM), and then was loaded into a cartridge. Then, an oil droplet (70 μL) was loaded into each cartridge, and the cartridge was covered with a gasket. Each cartridge was individually introduced into the droplet generator, and finally droplets of ~40 μL were transferred to the amplification plate. For each gene, three replicates were analyzed for each ethylene-treated sample and air-treated sample (control).

Primers for ddPCR were designed from cDNA sequences of the *G. imbricata* transcriptome (Table 1). PCR amplification was performed with a C1000 Touch Thermal Cycler (Bio-Rad) using the following conditions: an initial step at 95 °C for 5 min; followed by 40 cycles of 95 °C for 30 s, an experimentally determined annealing temperature (Table 1) for each gene for 1 min, 72 °C for 45 s; a single step at 4 °C for 5 min; and a temperature ramping from 4 °C to 90 °C at a rate of 2 °C s^−1^ for 5 min. After amplification, each sample was quantified using QuantaSoft v1.7.4 software (Bio-Rad). Data from merged wells (corresponding to each group of replicates) were retrieved, and the concentration of each group is given as the average number of transcript copies per μL.

### 4.5. Data Analysis

Gene expression (transcript copies per microliter) is reported herein as the mean ± standard deviation (SD). Statistical comparisons of concentrations were performed using R software (https://www.r-project.org; accesed on 20 May 2022). A one-way ANOVA followed by the post hoc tests Tukey HSD and Dunnett T3 was used to detect significant differences (*p* ≤ 0.01) between infertile thalli (at day 3) and thalli within early-stage cystocarp development (at day 10) with their respective controls.

## Figures and Tables

**Table 1:** Sequences of the forward (F) and reverse (R) primers for each gene involved in S-transporter and assimilation (*sulfate transporter*, *Sulfate adenylyltransferase*) as Carrageenan precursor (*phosphoglucomutase*, *galactose 1 phosphate uridylyltransferase*), in Carrageenan synthesis (*Galactose-6-Sulfurylases I and II*, *Carbohydrate sulfotransferase*), and in reproduction (*ODC*).

Gene	Primer Name	Sequence (5′-3′)
**S transporter and assimilation**		
*Sulfate transporter* *(S-transporter)*	ST-2545F ST-2545R	GGAAGATCCGGACGAGATTATG GGGTACCTTCGTCTAGTGTTTC
*Sulfate adenylyltransferase* *(SAT)*	SAT-790F SAT-790R	GAGGAATGCTGATGCTGTCT ACCTCGGTTAATGAGTTCTTCC
**Carrageenan precursor**		
*Phosphoglucomutase* *(PGM)*	PG-17368F PG-17368R	AGGTCGATAGCCGAGTTTAGA CCAGGATTGTGACTAGCTGTAAG
*Galactose 1 phosphate uridyltransferase* *(GALT)*	G1PU-1681F G1PU-1681R	GTAGTAGATGCCTGGTGTGATG CATATCTGGCCATGAGGATGAG
**Carrageen synthesis**		
*Galactose-6-Sulfurylase I* *(SYI.1)*	GS1-136F GS1-136R	ACAACGAGAAGGCTGACAAG CCGCACATTTGTTTCGCTATC
*Galactose-6-Sulfurylase I* *(SYI.2)*	GS1-824F GS1-824R	GAAACGGAGGTCACTCTTGTAG GAAGTCGACCGAGTTGCTTAT
*Galactose-6-Sulfurylase II* *(SYII.1)*	GS2-5356F GS2-5356R	GGAGGATTCTTGTTCGAGGATG AGTAGCGAGACCCGAGTATT
*Galactose-6-Sulfurylase II* *(SYII.2)*	GS2-6049F GS2-6049R	ATAACCCAAGTCCTCCTCCT GCTATCCCGTTCTTGCATCT
*Carbohydrate sulfotransferase* *(ST1)*	CS-3064F CS-3064R	CTGCATCACTGCGTTACTATTTC CATCAGGTCCAGCCACATAA
*Carbohydrate sulfotransferase* *(ST2)*	CS-3265F CS-3265R	TGTCGGGTGATGCGTTAAA TCGTTCCACTTTGTCCAGATAC
**Reproduction**		
*Ornithine decarboxylase* *(ODC)*	*D2-ODCF* *D2-ODCR*	5′3′ CGCAGACGCGACACAGTA 5′3′ TCACCAGAATGTTTAGCGAAGA
